# Video Consultations for Older Adults With Multimorbidity During the COVID-19 Pandemic: Protocol for an Exploratory Qualitative Study

**DOI:** 10.2196/22679

**Published:** 2020-10-26

**Authors:** Eng Sing Lee, Poay Sian Sabrina Lee, Evelyn Ai Ling Chew, Gayathri Muthulingam, Hui Li Koh, Shu Yun Tan, Yew Yoong Ding

**Affiliations:** 1 Clinical Research Unit National Healthcare Group Polyclinics Singapore Singapore; 2 Geriatric Education and Research Institute Singapore Singapore; 3 Department of Geriatric Medicine Tan Tock Seng Hospital Singapore Singapore

**Keywords:** COVID-19, multimorbidity, older adults, telemedicine, video consultation, elderly, telehealth, morbidity, protocol, chronic disease, high risk, qualitative

## Abstract

**Background:**

Multimorbidity, the coexistence of multiple chronic conditions in an individual, is a growing public health challenge. Amidst the COVID-19 pandemic, physical distancing remains an indispensable measure to limit the spread of the virus. This pertains especially to those belonging to high-risk groups, namely older adults with multimorbidity. In-person visits are discouraged for this cohort; hence, there is a need for an alternative form of consultation such as video consultations to continue the provision of care.

**Objective:**

The potential of video consultations has been explored in several studies. However, the emergence of COVID-19 presents us with an unprecedented opportunity to explore the use of this technological innovation in a time when physical distancing is imperative. This study will evaluate the sustainability of video consultations on a micro-, meso-, and macro-level by assessing the views of patients, physicians, and organizational and national policymakers, respectively.

**Methods:**

The NASSS (nonadoption, abandonment, scale-up, spread, and sustainability) framework was designed as a guide for the development of health care technologies. In this study, the implementation of and experiences related to video consultations will be studied using the NASSS framework. Individual in-depth interviews or focus group discussions will be conducted with participants using the Zoom platform. Data will be analyzed by at least two investigators trained in qualitative methodology, organized thematically, and coded in two phases—an initial phase and a focused selective phase. All disagreements will be resolved by consulting the larger research team until consensus is reached.

**Results:**

This study was approved for funding from the Geriatric Education and Research Institute. Ethics approval was obtained from the National Healthcare Group Domain Specific Review Board (reference #2020/00760). Study recruitment commenced in July 2020. The results of the data analysis are expected to be available by the end of the year.

**Conclusions:**

This study aims to evaluate the adoption and sustainability of video consultations for older adults with multimorbidity during the pandemic as well as post COVID-19. The study will yield knowledge that will challenge the current paradigm on how care is being delivered for community-dwelling older adults with multimorbidity. Findings will be shared with administrators in the health care sector in order to enhance the safety and quality of these video consultations to improve patient care for this group of population.

**International Registered Report Identifier (IRRID):**

DERR1-10.2196/22679

## Introduction

### Background

The utilization of remote consultations via telemedicine is not new to the health care scene [1]. Studies in several countries have discussed the benefits and challenges of remote consultations, which are often used to promote access to health care for rural and geographically isolated populations [2-5]. Other specific populations that have benefited from remote consultations are patients in palliative care [6,7], neonatal care [8,9] and postoperative care [10].

The COVID-19 pandemic has unexpectedly accentuated the need for remote consultations. In Singapore, the use of telemedicine apps has risen sharply [11], and dormitories housing foreign workers are using remote video technologies to monitor patients’ well-being during the pandemic [12]. Health care professionals in public primary care have started to defer in-person consultations and replace routine in-person follow-up appointments with telephone consultations. However, telephone consultations have serious limitations compared to in-person visits, since the clinician cannot use visual information about the patient to assess well-being. Video consultations offer a means of overcoming the limitation of not being able to see the patient, allowing for a more holistic assessment [1]. Despite the advantages of video consultation, its performance in terms of safety and effectiveness to render care for older adults with multimorbidity is unknown.

The potential of video consultations is of relevance to the geriatric population. Many older adults have multiple chronic conditions or multimorbidity [13], and require regular follow-up with different clinicians. As a high-risk group, older adults have been strongly encouraged to stay home during this crisis, as catching the infection poses serious risks for them [14]. Moreover, older adults with multimorbidity may face challenges related to physical mobility because of their ill health or frailty, making traveling to the clinic both dangerous and cumbersome. In order to maintain physical distancing, video consultations serve as a means for health care providers to continue care for this group of patients. To protect this vulnerable group of patients from COVID-19 infection, many of their scheduled physical medical appointments to primary care clinics have been postponed or converted to telephone consultations since February 2020 when Singapore moved into a semilockdown. Under the Ministry of Health’s regulatory sandbox for telemedicine providers, video consultation has been piloted in several private primary care clinics in Singapore since 2018 [15,16]. In the public sector, limited telemonitoring, telecollaborations, and telephone consultations have been used but video consultations have not been attempted.

In a recent online survey conducted at National Healthcare Group Polyclinics (NHGP) involving 712 patients who had telephone consultations in place of in-person consultations during the COVID-19 pandemic, approximately 95% of respondents agreed to use a telephone consultation again, and 93.1% would recommend it to another person. However, 8% of respondents preferred in-person consultations, citing a lack of personal touch as the main reason. Some of the respondents also complained that they could not see the doctor and would prefer a live video chat instead.

Conducting a video consultation may be considered a disruptive innovation since it simultaneously adheres to physical distancing measures during the COVID-19 pandemic while shifting the boundaries of doctor-patient consultations. Studies in other countries have shown that the implementation of telemedicine can be hindered by clinician nonacceptance and conflict with organization culture [17]. The successful introduction and implementation of video consultation will depend on the acceptability of the new intervention to both the intervention deliverers (ie, physicians) and the recipients (ie, patients). On a broader level, the health organizations’ readiness to adopt the innovation and the wider national context should also be taken into consideration [17].

Although Singapore is a nation with technologically developed infrastructure, levels of comfort with video technologies and tech savviness vary considerably. Communication issues including hearing and language difficulties may be exacerbated when the consultation is not performed in person, especially for older adults. Therefore, it remains to be seen what the benefits and drawbacks of a video consultation might be for older adults with multimorbidity in the Singapore context.

While the use of video consultations is promising as an alternative mode of care, extant literature has demonstrated the relevance of sociodemographic factors in patients with multimorbidity. Multimorbidity is known to be more prevalent in socioeconomically disadvantaged groups [18-20]; this was also evident in a study conducted in Singapore [13]. People from less privileged households in Singapore were reportedly less likely to have internet access or personal computers, according to Singapore’s Household Expenditure Survey 2017/2018 [21]. As such, the potential of video consultations for older adults with multimorbidity needs to take into consideration factors such as the digital divide leading to limited access among older adults, who tend to have lower levels of literacy and limited economic and social resources. While the present study acknowledges the ramifications of the digital divide, our current objectives are to pilot a video consultation workflow and identify challenges that even the more adept patients struggle with, as well as to identify whether there is a sizeable proportion of older adults with multimorbidity who are comfortable with video consultations. Being an IT (information technology)-related pilot project, we anticipate that some self-selection will occur, resulting in the patient population for this study potentially being more IT-literate than the general population.

The goal of this pilot project is to explore the adoption and sustainability of video consultations in order to arrive at a better understanding as to how the current health care system can be improved to ensure levels of care for patients can be maintained through this trying time. We also propose to use the findings acquired from the study as a precursor to further studies that will inform the future implementation of video consultation with family physicians for older adults with multimorbidity in the primary care setting in Singapore.

### Objectives

The objectives of this study are as follows:

1. At the micro-level (individual technology users): to study how patients are seen, heard and their needs are met in a safe manner; to explore patients’ and physicians’ acceptance, communication issues and logistical demands, thereby elucidating the features of an optimal video consultation
2. At the meso-level (organizational processes and systems): to understand and explore the social, technical, financial, and logistical support or lack thereof from the organization, thereby elucidating the requirements for introducing, sustaining, and scaling up of video consultations
3. At the macro-level (national policy and wider context): to understand the current regulatory, legal, professional, sociocultural, political, or policy context, in order to alert key stakeholders to the potential barriers and challenges of video consultation as a regular health care service model in the post–COVID-19 phase.

### Research Questions

Our research questions are as follows:

1. What features characterize an optimal video consultation and how does the experience differ from an in-person consultation?
2. On an organizational level, what are the factors that affect the implementation of video consultations?
3. What is the national-level context of implementing video consultations as a regular health care service model option in the post–COVID-19 new normal?

## Methods

### Study Design and Conceptual Framework

This is a predominantly qualitative descriptive study for understanding the perspectives of various stakeholders (patients, family physicians, organizational implementers/leaders, and organizational/national policymakers) on video consultation for community-dwelling older adults with multimorbidity in a public primary health care setting in Singapore.

**Figure 1:**
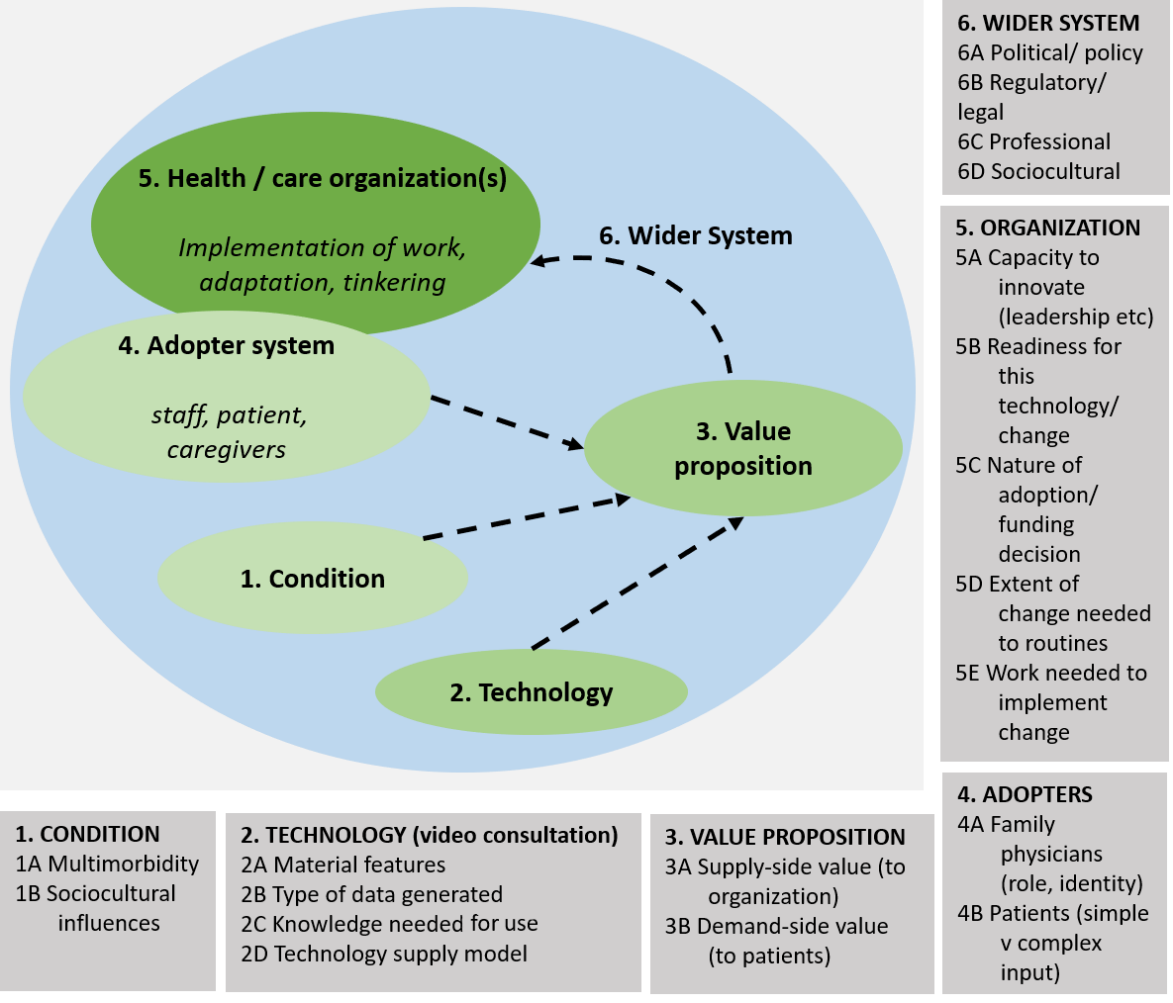
The NASSS (nonadoption, abandonment, scale-up, spread, and sustainability) framework (adapted from Greenhalgh et al [17]).

Six domains from the nonadoption, abandonment, scale-up, spread, and sustainability (NASSS) technology implementation framework [17] will be adopted to guide our study design and analysis (Figure 1). The domains are the health condition, the technology, the value proposition to patients, the adopter system (family physician and patient), the health care organization (including attention to implementation and adaptation), and the wider system (related political, regulatory, legal, professional, and sociocultural factors). We propose to use the framework prospectively and in real time to explore the challenges of introducing and implementing video consultations to support a new health care model.

### Setting

All provision of health care services in Singapore is based on the fee-for-service model with various forms of subsidies available for those in need. The government often finetunes the subsidy policies to regulate “the supply and prices of health care services in the country” to keep public sector health care costs in check. However, private medical care is largely subject to market forces.

Singaporeans not empaneled to any specific practice and are free to choose the providers within the government or private health care delivery system. The public primary health care services are provided through an island-wide network of 20 outpatient polyclinics, which provide subsidized primary care including primary medical treatment, preventive health care, and health education. In addition, there are up to 1700 clinics run by private general practitioners. For this study, recruitment will be based at NHGP, which consist of six polyclinics located in the northern and central parts of Singapore.

### Sampling and Recruitment of Participants

Purposive sampling will be used to obtain views from four participant groups for this study: patients, family physicians, organizational implementers/leaders, and organizational/national policymakers. The patient group will include older adults aged 60 years and above with multimorbidity (at least three chronic conditions) being managed by the polyclinic who have undergone at least one video consultation. The family physician group will include family physicians who have conducted at least two video consultations. The organizational implementers/leaders will include members of the senior management team, family physician leaders, or operational staff of NHGP involved in the pilot video consultation project. Lastly, the organizational/national policymakers will include office holders from NHGP and relevant statutory boards or government departments.

Potential patient participants will be approached when they visit the clinic for their laboratory tests. Routinely, an in-person consultation will follow one week after their laboratory tests. Patients who are eligible and comfortable to convert their impending in-person visit to video consultation will be referred to a research team member after their laboratory tests. Written informed consent will then be obtained from patients by a research team member in clinic after explaining the study and providing ample time for them to ponder and decide. A convenient time for an individual in-depth interview will be arranged within a week after the scheduled video consultation. All nonpatient participants will be sent an email invitation with information on the study. Written informed consent for nonpatient participants will be obtained by a study team member at their workplace. All study participants will be informed that the interviews (family physicians and organizational implementers/leaders) or focus group discussions (organizational/national policymakers) will be conducted using an institution-approved secure video conferencing platform and recorded using a digital audio recorder.

An estimated maximum number of 60 participants will be recruited: 20 patients (aged ≥60 years with at least three chronic conditions), 15 family physicians, 15 organizational implementers/leaders, and 10 organizational/national policymakers. The recruitment of participants may end earlier if the interview data have reached thematic saturation.

### Data Collection

Individual in-depth interviews and focus group discussions will be conducted by research team members who have experience in qualitative research. Each interview and discussion is expected to last up to 40 minutes and 90 minutes, respectively. They will be conducted via the institution-approved secure Zoom platform [22,23]. The Zoom platform as a tool to conduct video interviews allows for physical distancing to be maintained so that the research project can be conducted during the COVID-19 pandemic period. The topic guides are developed from the NASSS and complexity assessment tool (CAT) framework. They include questions on the micro-level (patients and physicians), the meso-level (organizational implementers/leaders), and the macro-level (organizational/national policymakers) [24].

Questions at the micro-level will pertain to patients’ and physicians’ experiences of the video consultation (acceptability, communication needs, safety, and related logistics). At the meso-level, questions put to organizational implementers/leaders will relate to discovering what factors and processes within the organization contribute to or hinder the successful execution of video consultations. Finally, questions at the macro-level put to organizational/national policymakers will seek to grasp the relevant regulatory, legal, professional, and sociocultural context in which the innovation is implemented, to better understand what challenges may lie ahead if video consultations become a regular service model nationwide. We will be collecting demographic information from patients, including questions on household type, people they live with, and level of IT literacy. We will also engage the family physicians in structured reflective journaling, where they will be asked to provide reflections on their video consultation experiences. This will occur at three different time points (at the beginning of their involvement in the study, midway through the study period, and at the end of the study), allowing the study team members to understand how their views change over the course of initiating and conducting video consultations.

### Data Analysis

The interviews and focus group discussions will be transcribed verbatim. Another data source will include the reflective journals of the family physicians. Inductive thematic analysis will be carried out by at least two investigators trained in qualitative methodology [25]. They will be working independently initially and develop preliminary coding structures for organizing the data thematically. Coding would be done in two phases—an initial phase and a focused selective phase. Once the coding scheme has been refined, relationships among categories will be explored to facilitate raising the analytical level from categorical to thematic in order to make meaningful interpretations of the data. All disagreements will be resolved by consulting the larger research team. NVivo version 12 Plus (QSR International) will be used to help organize the data.

### Rigor

Credibility will be achieved through obtaining data from all stakeholders and investigator triangulation [26]. We aim to achieve transferability by providing a clear description of the participants, settings, and research process [27]. Dependability will be maintained by keeping field notes and recording all analytic decisions. Finally, we aspire to achieve confirmability by applying data triangulation and researcher reflexivity [26].

### Ethics and Confidentiality

We have obtained ethics approval from the National Healthcare Group Domain Specific Review Board (reference #2020/00760). Study team members will ensure that the necessary precautions are taken to ensure that privacy and confidentiality are maintained, such as using institution-approved secure Zoom accounts that are password-protected and accessible by study team members only.

## Results

This project received funding from the Geriatric Education and Research Institute under the COVID-19 grant. At the time of writing, no participants have been formally recruited, but clinicians have been contacted and invited to take part in the study. Literature review is ongoing for researchers to stay updated on the state of the field in telemedicine research. Given that this study is qualitative and exploratory, it would be precipitous to anticipate our findings in detail. However, we expect a diversity of opinions both within and between the patient, physician, organizational implementer/leaders, and policymaker groups. The patient and physician groups are likely to show a range of opinions that spans from enthusiasm to reluctance, although the specific reasons for these will need to be elucidated. Organizational implementers/leaders are likely to discuss operational and logistical demands, and their impact on the operation of the whole organization, while we expect policymakers to help us frame the views within a larger overarching context. It is precisely this detail and the divergence in opinions among different stakeholders that will enable us to grasp what challenges lie ahead for the eventual successful implementation and large-scale adoption of video consultation as part of routine care for older adults with multimorbidity.

## Discussion

This qualitative study is intended to understand how video consultation can benefit both older adults with multimorbidity and family physicians in Singapore. This is the first study looking into using video consultation in public primary health care in the country.

The most recent NHS England long-term plan mandates the availability of video consultations in the next 5 years [28]. Comparing video consultations to telephone consultations, it has been shown to have a better rapport to facilitate understanding through nonverbal communication [28]. Video consultations have been found to support patients with chronic conditions and prevent unnecessary admissions to long-term institutional care [29]. In Canada, they have made possible an interprofessional model of practice involving hospital specialists, a social worker, a pharmacist, a home care community coordinator, and other allied health professionals who allow older patients with complex multimorbidity to be managed within the community [30]. Jiwa et al [31] reported that Australian general practitioners who were favorably inclined toward video consultations were more likely to work in larger practices and be more established professionally. Approximately one-third of them were positive about video consultations, one-third were ambivalent, and one-third were against them.

The COVID-19 crisis has led to the introduction of video consultations in Singapore’s public primary health care setting. If found to be appropriate for future use, this study will offer practical guidance on maximizing the adoption of this consultation model for desired clinical outcomes and user acceptance.

Before scaling and spreading video consultations in primary care, all stakeholders should be involved in further studies, which will look at proof of concept for clinical outcomes, patient suitability, safety, and minimization of technical issues by referencing qualitative findings from this study; and proof of value for the cost-effectiveness of the model of care. Finally, for the implementation and scaling phases of video consultations, clinical process re-engineering is likely to be necessary to create the clinical, quality educational, business, logistic, and organizational systems necessary to support implementation of video consultations on an enterprise scale.

As mentioned above, the digital divide is a reality in Singapore despite its affluence and high levels of general literacy and technological literacy. Subsequent phases looking into full-scale implementation will certainly need to examine the practicability and desirability of making video consultations available to all sociodemographic sectors, providing extra IT support, taking into account a lower level of technological access and literacy among the elderly in economically less-advantaged areas. This future phase will need to examine the specific role video consultations can play in future health care models, whether in supplementing routine consultations or enabling doctors to connect with patients with mobility issues, for example. The present study thus represents only an initial step toward a much larger initiative involving the role of video consultations within the health care system in primary care.

## Appendix

Multimedia Appendix 1 Peer Review Report from the Geriatric Education and Research Institute.

## Abbreviations

CAT: complexity assessment tool
GERI: Geriatric Education and Research Institute
IT: information technology
NASSS: nonadoption, abandonment, scale-up, spread, and sustainability
NHGP: National Healthcare Group Polyclinics
