# Supplementary material for: Video Consultations for Older Adults With Multimorbidity During the COVID-19 Pandemic: Protocol for an Exploratory Qualitative Study
Source: JMIR Res Protoc. 2020 Oct 26;9(10):e22679. doi: 10.2196/22679 (PMC7592461; doi:10.2196/22679)
Supplement: Multimedia Appendix 1 [file resprot_v9i10e22679_app1.pdf]

Geriatric Education & Research Institute

APPENDIX A

Reviewers' Comments

1. The application was reviewed by four independent reviewers and their comments are compiled below.

Review Criteria	Average Score
Relevance (0.0 to 10.0)	9.00
Repercussions (0.0 to 20.0)	15.50
Rigor (0.0 to 20.0)	15.50
Readiness (0.0 to 10.0)	9.00
Reasonableness (0.0 to 10.0)	9.00
Total Score (Max = 70)	58.00
**Comments**	
1. Well written with a comprehensive view from micro to macro level. Good translation potential Applying IoT method is innovation but need social scientist on team familiar with topic of social isolation<br>2. Did not present any evidence on efficacy of video consultations in disease management.<br>3. Research Question 3 can be more clearly framed<br>4. Given the inductive nature of analysis, good to state upfront how the NASSS conceptual framework will be applied.<br>5. Self-select group of older adults >60 years who can use ZOOM which may not reflect the general population. That is an inevitable weakness of the study which must be acknowledged.	
